# Supplementary material for: Fingerstick blood assay maps real‐world NAD + disparity across gender and age
Source: Aging Cell. 2023 Aug 28;22(10):e13965. doi: 10.1111/acel.13965 (PMC10577551; doi:10.1111/acel.13965)
Supplement: Supplementary file 1 — Data S1. [file ACEL-22-e13965-s001.docx]

**Fingerstick blood assay maps real-world NAD^+^ disparity across gender and age**

Authors: Pei Wang^1#^, Meiting Chen^1#^, Yaying Hou^2#^, Jun Luan^3^, Ruili Liu^2^, Liuqing Chen^1^*, Min Hu^3^*, Qiuliyang Yu^1^*

**Affiliations:**

1 Sino-European Center of Biomedicine and Health, Shenzhen Key Laboratory for the Intelligent Microbial Manufacturing of Medicines, Shenzhen Institute of Advanced Technology, Chinese Academy of Sciences, 518055 Shenzhen, China

2 Celfull (China) Operation and Research Center, 518063, Shenzhen, China

3 Department of Sports Medicine, Guangzhou Sport University, 510150 Guangzhou, China

^#^ Equal Contribution.

* Correspondence: Liuqing Chen: [lq.chen@siat.ac.cn](mailto:lq.chen@siat.ac.cn), Min Hu: [whoomin@aliyun.com](mailto:whoomin@aliyun.com), Qiuliyang Yu: [q.yu@siat.ac.cn](mailto:q.yu@siat.ac.cn)

Lead contact: Qiuliyang Yu

**Table S1. Age and gender information of subjects for evaluating NMN supplementation without sport.**

|  | Placebo | 500 mg NMN | 1000 mg NMN |
| --- | --- | --- | --- |
| Sample size | 25 | 25 | 25 |
| Demographic | Han Chinese (25) | Han Chinese (25) | Han Chinese (25) |
| Dropout | 0 | 6 | 4 |
| Gender (M/F) | 10/15 | 9/10 | 10/11 |
| Average age | 57.00 ± 6.86 | 57.95 ± 5.23 | 58.10 ± 4.22 |
| Aerobic sport (Yes/No) | No | No | No |
| Average NAD^+^ | 23.8 ± 5.5 | 41.7 ± 13.0 | 58.8 ± 21.1 |

**Table S2. Age and gender information of subjects for evaluating NMN supplementation in combination with moderate level of sport.**

|  | Placebo | 500 mg NMN |
| --- | --- | --- |
| Sample size | 21 | 21 |
| Demographic | Han Chinese (21) | Han Chinese (21) |
| Dropout | 0 | 1 |
| Gender (M/F) | 10/11 | 10/10 |
| Average age | 58.21 ± 5.39 | 58.59 ± 4.75 |
| Aerobic sport (Yes/No) | Yes | Yes |
| Average NAD^+^ | 33.18 ± 7.18 | 55.48 ± 21.37 |

**Table S3. Cost comparison of NAD^+^ quantification methods**

| Method | Device | Device cost (USD) | Running cost/sample (USD) | Measurement time (min) | Direct measurement (Y/N) |
| --- | --- | --- | --- | --- | --- |
| HPLC-MS | HPLC-MS | Around 100,000 | 10-15 | 10-15 | Yes |
| NADH/NAD^+^ assay kit | Plate reader | 20,000-40,000 | 10-15 | 45-90 | No |
| Bioluminescent sensor | NAD^+^ analyzer | Around 10,000 | Less than 5 | 3-5 | Yes |

**Table S4. Age and gender information of subjects for surveying NAD^+^ level via fingerstick sample**

| Age range | 20-50 | | 50-85 | |
| --- | --- | --- | --- | --- |
| Gender | M | F | M | F |
| Sample size | 24 | 23 | 46 | 56 |
| Average age | 33.42 ± 7.02 | 33.35 ± 8.56 | 67.88 ± 9.33 | 67.23 ± 8.55 |
| Average NAD^+^ | 44.2 ± 18.9 | 32.7 ± 9.6 | 25.9 ± 9.8 | 24.8 ± 9.6 |

**
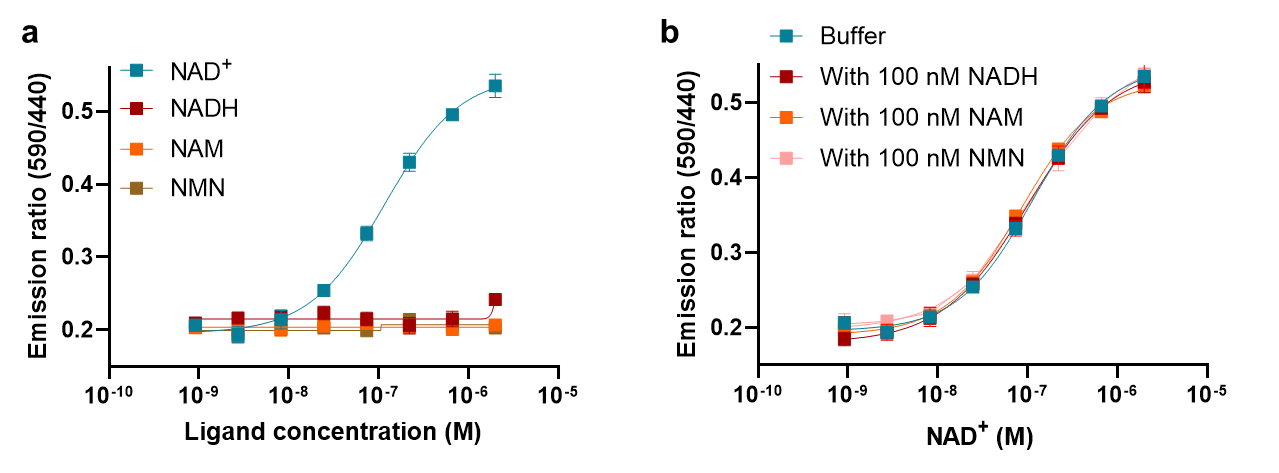
Figure S1. Specificity and resistance to interference of** **NS-Goji 1.3. (a)** Response of NS-Goji 1.3 towards NAD^+^, NADH, NAM and NMN. **(b)** Response of NS-Goji 1.3 towards NAD^+^ in the presence of NADH, NAM or NMN. NADH, NAM and NMN levels are below 100 nM in the measurement buffer after the sample preparation procedure for NAD+ measurement. Error bars represent SD of n = 3 independent measurements.


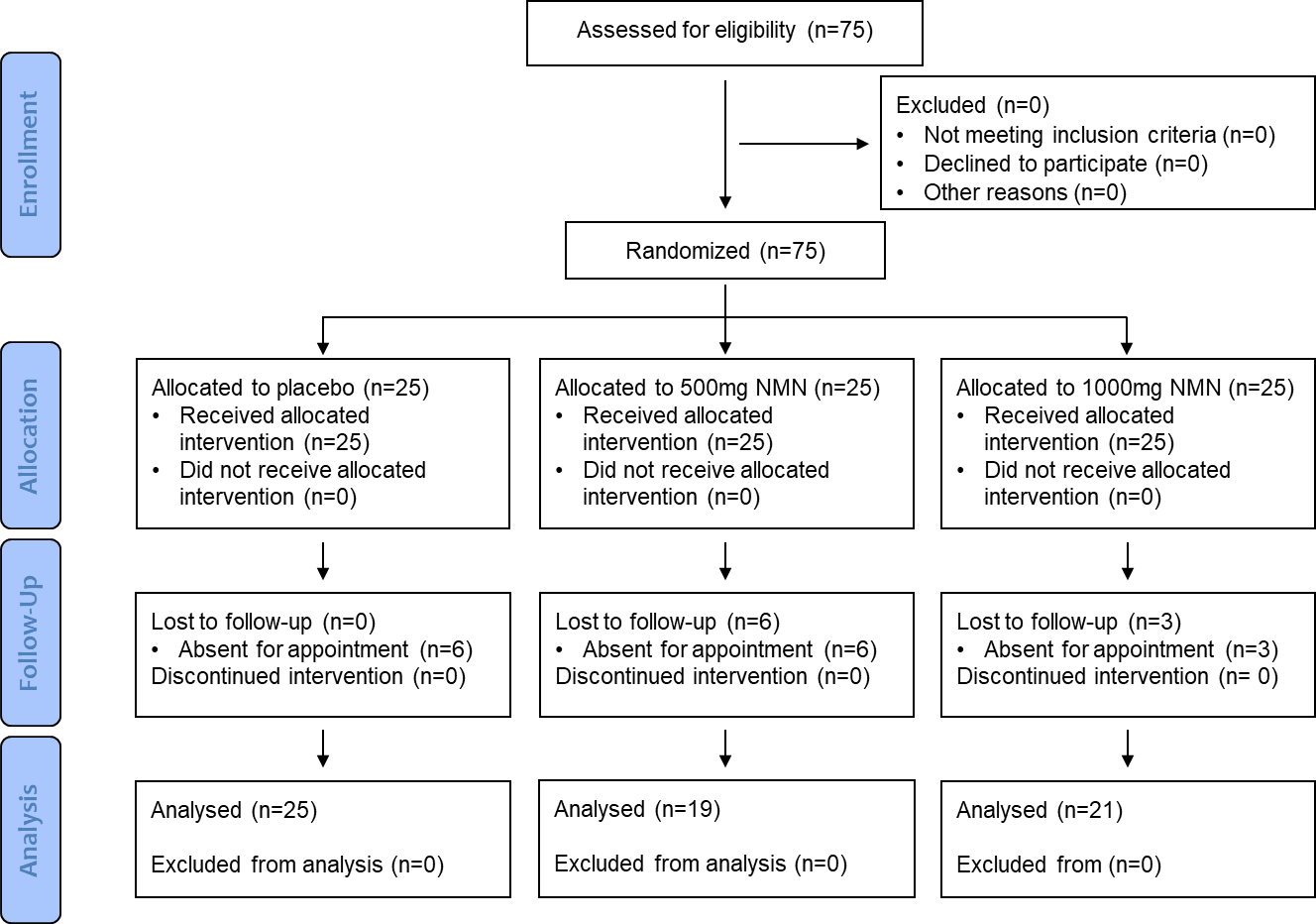


**Figure S2. CONSORT flow diagram of clinical study of NMN supplementation.**

**
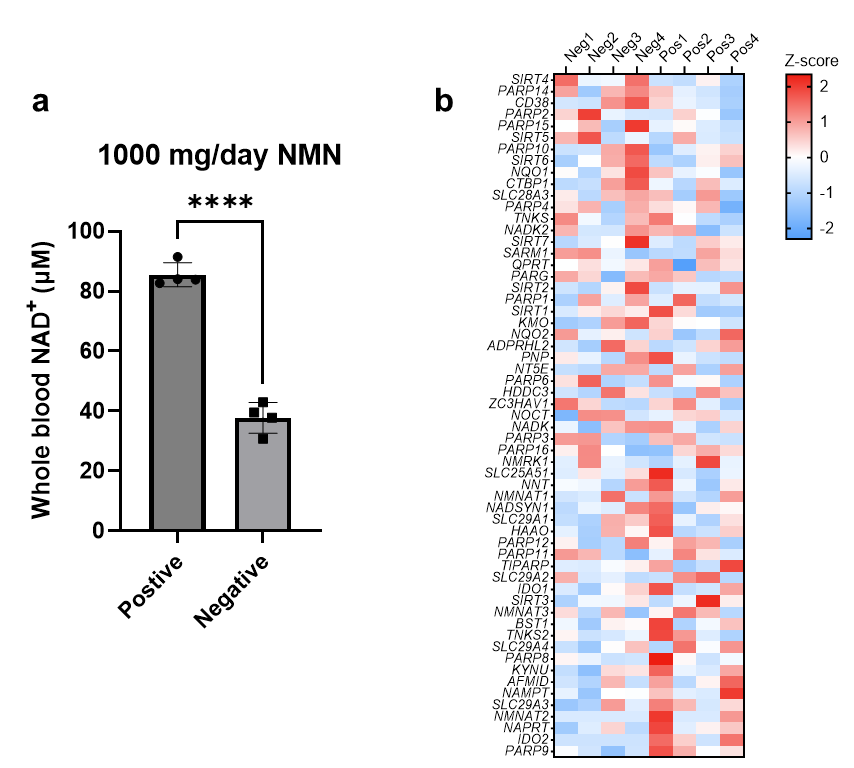
**

**Figure S3. Relative expression level of NAD^+^ metabolism-related genes for subjects responsive and inert to NMN supplementation.** **(a)** Whole blood NAD+ levels measured from NMN responders and non-responders after 30 days of 1000 mg/day NMN supplementation. **(b)** The expression profile of NAD^+^ metabolism-related genes were obtained by sequencing PBMCs isolated from venous blood at the end of the clinical study for 4 subjects responsive and inert to NMN supplementation based on measured NAD^+^ levels. Error bars represent SD, significance was calculated using t-test for (a), **** *p* < 0.0001.

**
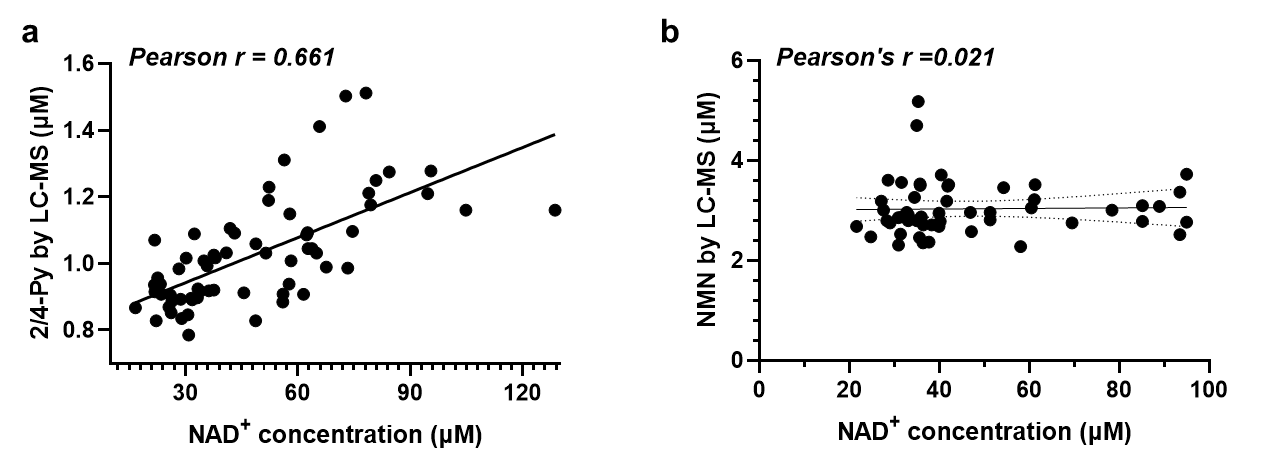
Figure S4. Correlation between NAD+ and metabolites in clinical samples. (a)** Correlation between whole blood 2/4-Py and NAD^+^ with *Pearson’s r* = 0.661. **(b)** Correlation between whole blood NMN and NAD^+^ with *Pearson’s r* = 0.021.


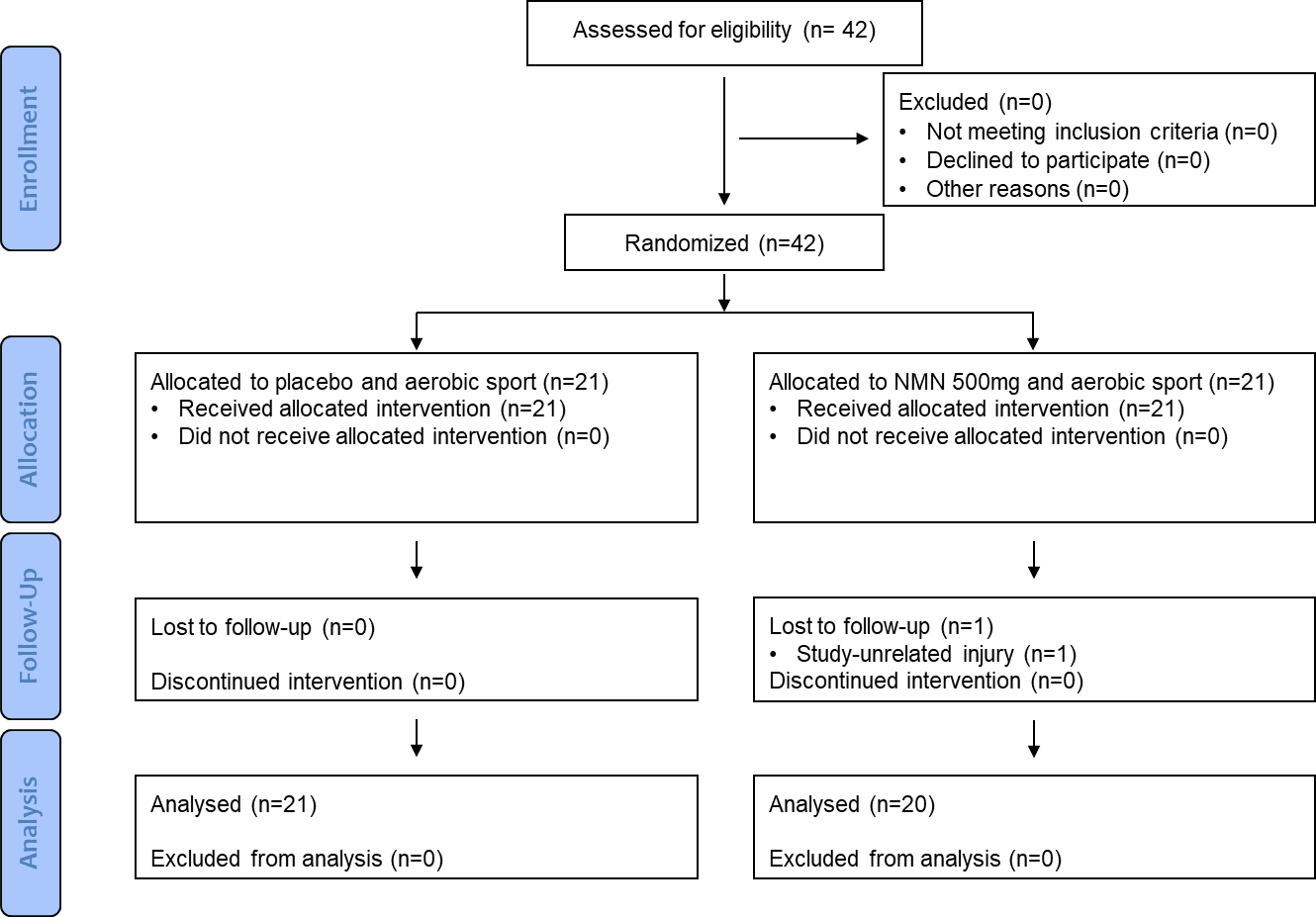


**Figure S5. CONSORT flow diagram of clinical study of NMN supplementation with aerobic sport.**


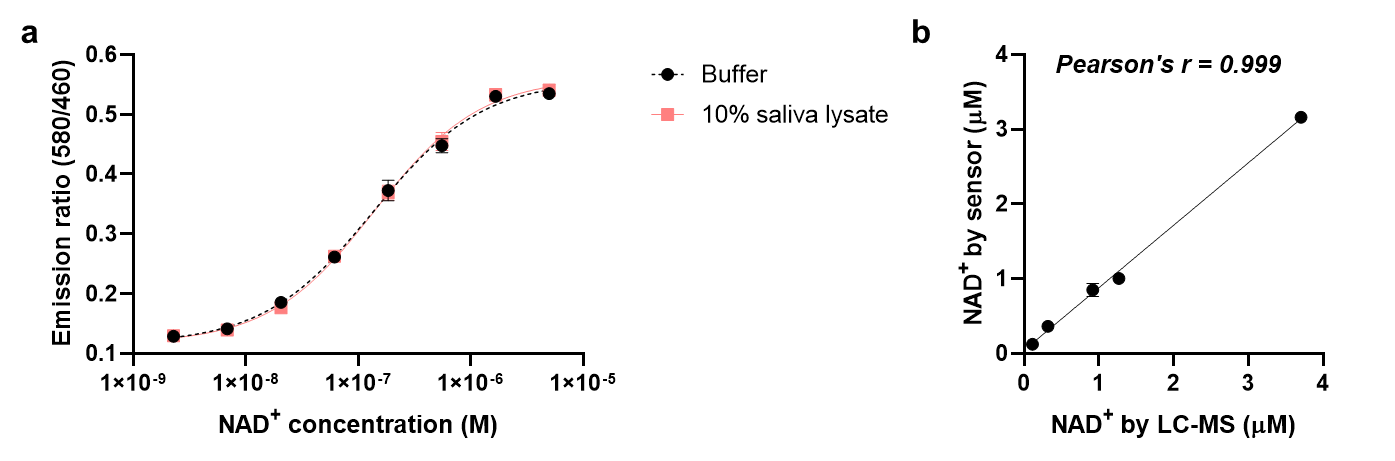


**Figure S6. Measurement of NAD^+^ in spiked saliva samples. (a)** Sensor titration curve in normal buffer and buffer spiked with 10% saliva lysate. The presence of saliva lysate did not show interference with the sensor performance. **(b)** Comparison between sensor and LC-MS measurement of NAD+ in saliva. The two methods showed a high level of agreement with Pearson’s r = 0.999. In (a) and (b), values are given as mean ± SD of three independent measurements.

**
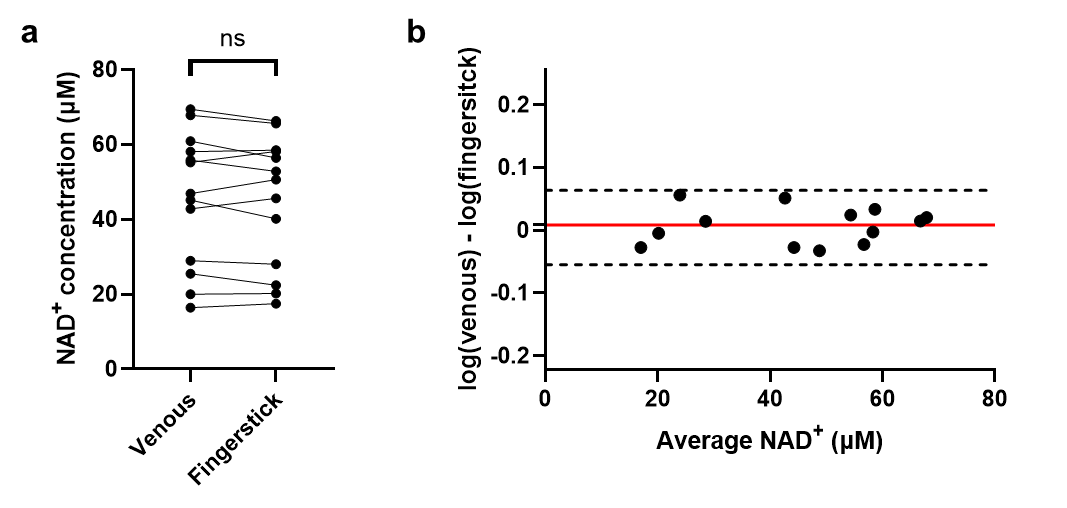
Figure S7. Correlation between fingerstick and venous NAD^+^. (a)** Comparison of NAD^+^ levels measured from venous and fingerstick samples. No significant difference was found between the two sample types. Significance was determined using paired t-test. **(b)** Bland-Altman analysis for venous NAD^+^ measured from venous and fingerstick blood samples.

**Figure S8. Degradation of NAD^+^ in biological samples at room temperature and 4 ^◦^C measured by LC-MS.** Storage at room temperature induced considerable degradation of NAD^+^ in blood samples compared to the storage at 4 ^◦^C. Error bars represent SD of n = 6 independent biological repeats.
